# Surgical Removal of Numerous Foreign Body Gastric Bezoar: A Case Report

**DOI:** 10.7759/cureus.4173

**Published:** 2019-03-04

**Authors:** Mohamed Ahmed, Saba Habis, Ahmed Mahmoud, Rasha Saeed, Mohamed Elkahly

**Affiliations:** 1 Surgery, Riverside Community Hospital, Riverside, USA; 2 Internal Medicine, Riverside Community Hospital, Riverside, USA

**Keywords:** gastric outlet obstruction, bezoar, mental retardation

## Abstract

A 53-year-old mentally retarded male was brought to our emergency room after vomiting a plastic glove. Computed tomography revealed marked gastric distention containing large amount of residual food debris. Endoscopic retrieval was unsuccessful. Surgical removal of the foreign bodies was done. The patient did well and was discharged from the hospital.

## Introduction

Gastric bezoars are foreign bodies in the stomach that increase in size due to accumulation of nonabsorbable food or fibers [1]. It is defined as “any of various calculi found chiefly in the gastrointestinal organs and formerly believed to possess magical properties from Arabic bāzahr” [2]. The clinical picture ranges from abdominal pain, nausea, vomiting, early satiety and weight loss, to bleeding ulcer, intestinal obstruction and perforation [3].

## Case presentation

**Figure 1:**
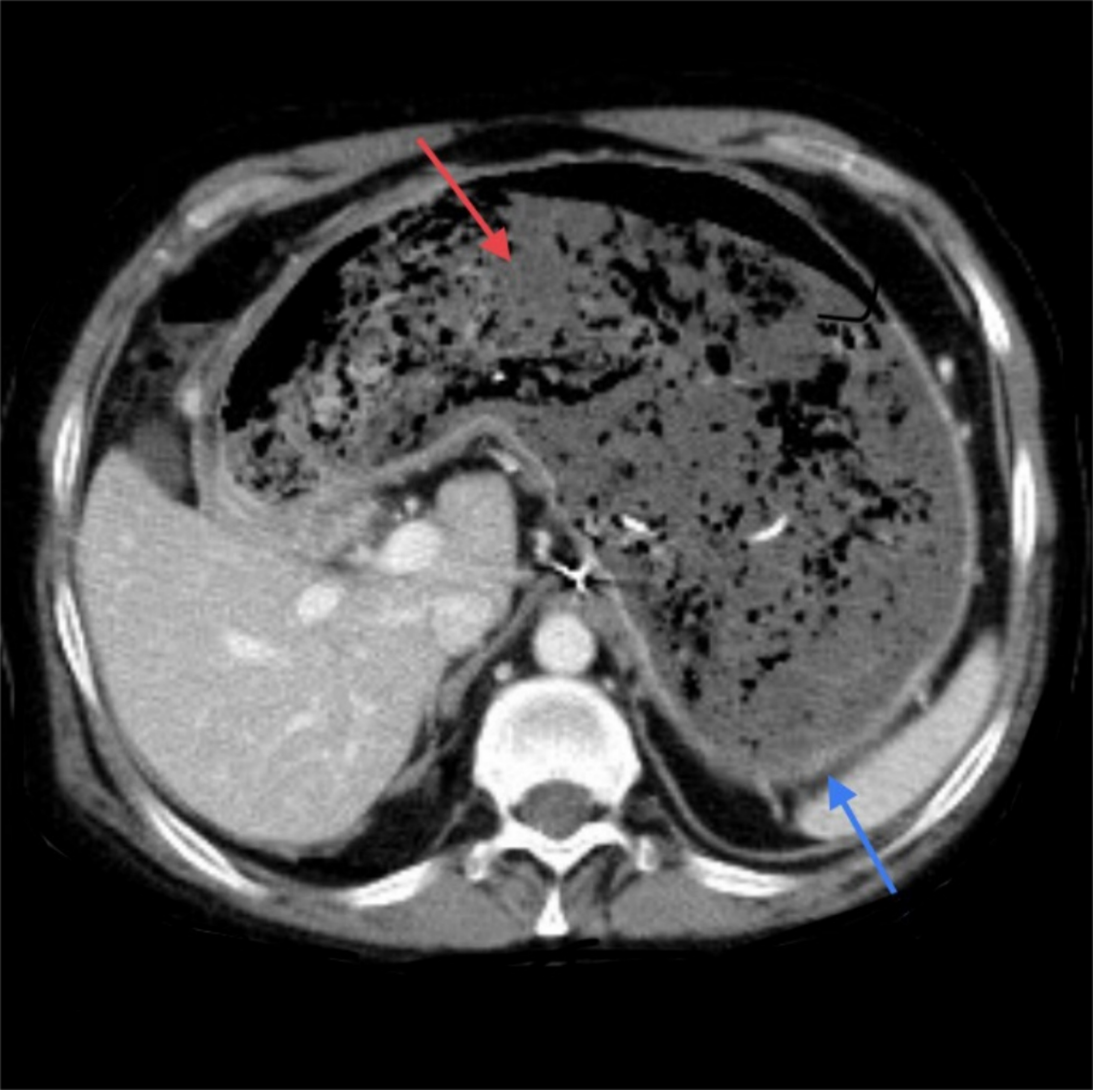
Computed tomography (CT) scan abdomen. Stomach (blue arrow). None enhancing mass (red arrow).

A 53-year-old mentally retarded male was brought to our emergency room after vomiting a plastic glove. The patient had two weeks history of intermittent nausea and vomiting. Computed tomography of the abdomen showed a non-enhancing mixed density intraluminal gastric mass (Figure 1).

**Figure 2:**
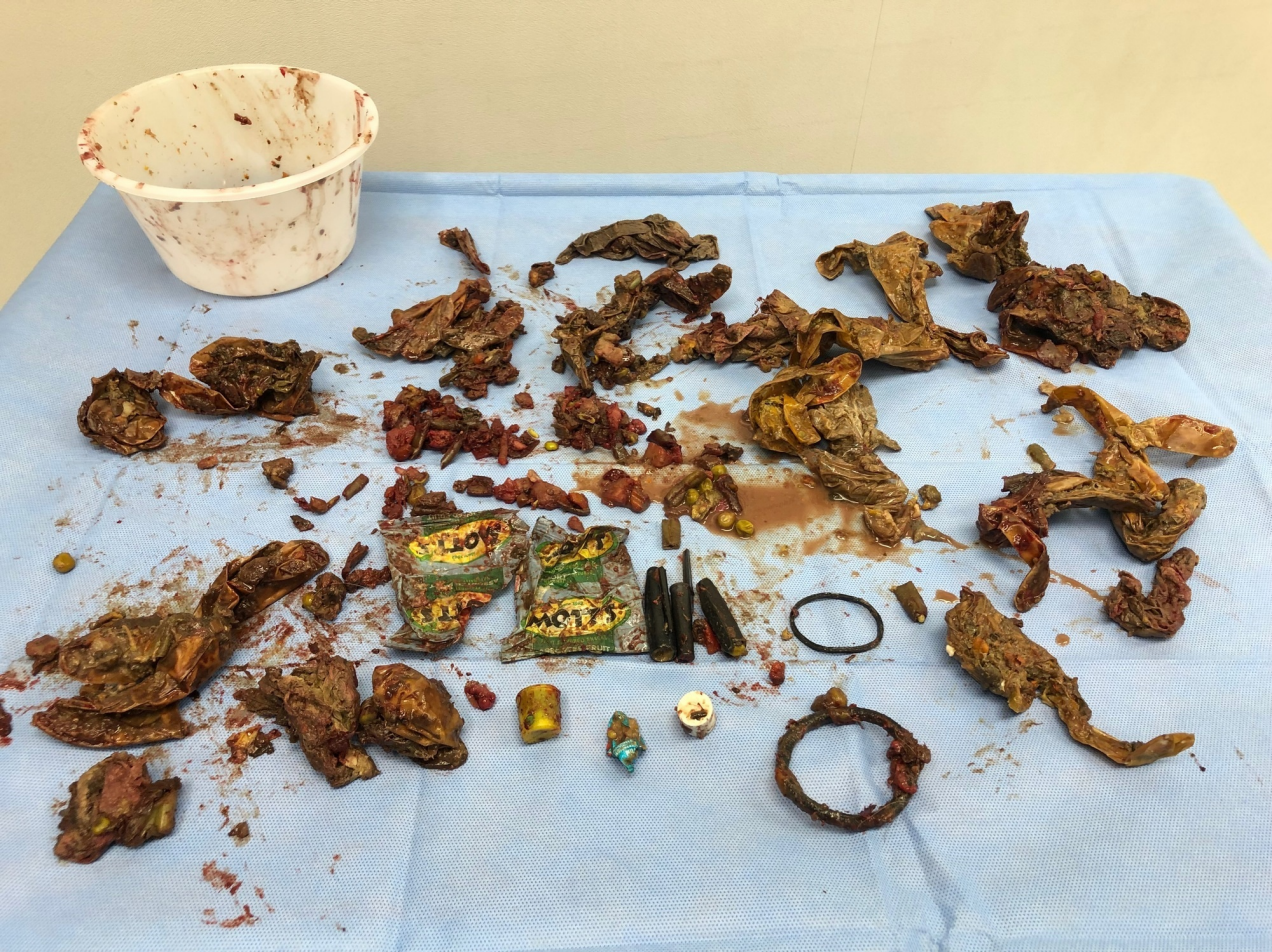
Stomach content. Variety of foreign bodies retrieved.

An endoscopy done to confirm the diagnosis and retrieve the foreign bodies was not successful. The patient was taken to the operating room and an upper midline incision was done (multiple previous abdominal surgeries). Gastrotomy and foreign body bezoars removal was performed (Figure 2).

The patient did well and was discharged from the hospital.

## Discussion

More than 90% of bezoars cases are found in children and young female with pica, psychiatric disorders, or mental retardation [4], but rarely a severe psychiatric disorder is seen [5]. The clinical picture ranges from abdominal pain, nausea, vomiting, early satiety and weight loss, to bleeding ulcer, intestinal obstruction and perforation. Anemia and hypoalbuminemia associated with chronic gastritis usually go unnoticed until the case is brought to light by the onset of more severe complications such as hemorrhage, enteric or pancreatic or biliary obstruction [6]. Diagnostic modalities include abdominal ultrasound (US), computed tomography (CT) scan and upper endoscopy. CT scan has a higher accuracy rate than US [7]. Treatment of bezoars includes observation, dissolution, fragmentation, laparotomy or laparoscopic gastrotomy for removal. Gastroscopic fragmentation, nasogastric lavage or suction, and enzymatic therapy with cellulose and papain have been tried [8]. In our case, the use of endoscopy was not successful and surgical retrieval was the best option.

## Conclusions

Bezoars are a potentially serious problem. Treatment includes gastric lavage, dissolution, endoscopic retrieval and surgery. It is prudent to prevent future occurrences via dietary counseling, avoidance of certain medications, and correction of underlying motility disorders if present. After successful management, psychiatric evaluation should be considered and the patient and/or his care giver should be educated to prevent recurrence.
